# Assessment of Intraocular Measurements in Neonatal Foals and Association with Gender, Laterality, and Body Weight: A Clinical Study

**DOI:** 10.1371/journal.pone.0109491

**Published:** 2014-10-08

**Authors:** Simona Valentini, Carolina Castagnetti, Vincenzo Musella, Giuseppe Spinella

**Affiliations:** 1 Department of Veterinary Medical Sciences, University of Bologna, Ozzano dell'Emilia (BO), Italy; 2 Department of Health Sciences, University Magna Graecia of Catanzaro, Germaneto (CZ), Italy; University of Rennes 1, France

## Abstract

Objective of this study was to describe intraocular measurements in newly born foals (1–7 days of age) and assess the association between globe measurements and gender, laterality, and body weight. B-scan ultrasonographic biometry was performed on both eyes of 22 healthy foals (44 eyes) ages 1–7 days using a 10-MHz transducer. Intraocular measurements (anterior chamber depth, central lens thickness, vitreous chamber depth, axial globe length, longitudinal globe length, lens poles distance) were carried out using the ultrasound internal calipers. The influence of gender (male or female), laterality (right or left eye), and body weight (“light” <48 kg; “heavy” ≥48 kg) on ocular measurements was analysed by the Student *t* test. Values of P<0.05 were accepted as significant for all analyses. Mean anterior chamber depth was 2.2±0.5 mm (Standard Deviation); central lens thickness was 9.9±0.8 mm; vitreous chamber depth was 15.5±1.1 mm; axial globe length was 27.6±1.6 mm; longitudinal globe length was 35.8±1.2 mm, and lens poles distance was 16.4±1.0 mm. Intraocular measurements were not influenced by gender, laterality nor body weight. This study provides reference values for intraocular measurements in neonatal foals and may be useful in the diagnosis and treatment of congenital and acquired pathologies involving the globe.

## Introduction

In equine medicine, a complete ophthalmic examination is sometimes limited by the opacification of the anterior segment and lens, making it difficult to correctly diagnose ocular disease. Ultrasonography provides the advantage of a complete image of the globe in awake animals regardless of globe opacities and provides excellent morphologic details of the globe (size, shape, and position), intraocular structures, retrobulbar space, and bony orbit [1], [2]. The technique is safe and practical, requiring only topical anaesthesia and sometimes sedation [1], [2]. However, knowledge of the sonographic intraocular measurements of a normal eye is essential for a correct diagnosis of ocular diseases, as well as to screen candidates for cataract surgery and intraocular lens implantation [3], [4].

Few reports exist regarding the axial dimensions of globes and intraocular distances measured via B-scan ultrasonography in the adult horse [3]–[6]. Even fewer reports exist regarding ultrasonographic biometry of the eye in the neonate, despite the relatively high number of acquired and congenital ophthalmic lesions diagnosed in healthy foals within 48 hours of life [7], [8]. Townsend et al. examined foals aged more than 42 days [9], and Grinniger et al. performed ultrasonographic biometry on 33 eyes of horses aged 0–2.5 years but did not specify the age distribution of subjects [5]. To the authors’ knowledge, there are no reference values available in veterinary medical literature for intraocular distances such as anterior chamber depth (ACD), central lens thickness (CLT), lens pole distance (LPD), vitreous chamber depth (VCD), axial globe length (AGL), and longitudinal globe length (LGL) in neonatal foals (from 1–7 days of age).

The aim of this study was to report ultrasonographic measurements of globes in neonatal foals and to determine the effect of gender, laterality, and body weight on these parameters, in order to establish reference values that could function as a tool to discriminate between normal and abnormal conditions.

Our hypothesis was that ultrasonographic ocular measurements in newly born foals are smaller than values reported in the literature for older foals and could not be influenced by variables such as gender, laterality, and body weight.

## Methods

### Animals

Twenty-two foals of various breeds were included in the study; age distribution was 1–7 days.

All horses were submitted to brief ophthalmic examination: the eyes were examined in ambient barn light for gross abnormalities and a direct examination in a dark area with a direct ophtalmoscope was performed. Individuals with ocular abnormalities were excluded from the study. Breed and age of each foal were recorded; body weight was measured by a weight tape. All procedures on the animals were carried out with the approval of the Ethical Committee of the University of Bologna (Prot. 8134-X/10 02/22/2011), in accordance with DL 116/92, approved by the Ministry of Health. Oral informed consent was given by the owners.

### Ocular measurements

B-scan ultrasonographic biometry (MyLab One, ESAOTE, Genova, Italy) was performed on both eyes of each foal. A 10-MHz linear probe was used to obtain all images using a transcorneal approach; ACD was measured using a small, custom-made stand-off to minimize compression of the globe. LGL alone was measured with a 7.5-MHz microconvex array. For all measurements, foals were manually restrained with the head maintained in a normal, upright position. Auriculo-palpebral nerve blocks were not performed, and sedative drugs were not administered. Prior to data collection, 0.2 mL oxibruprocaine hydrochloride 1% ophthalmic solution (Novesina, Novartis, Orrigio, Italy) was applied to the corneal surface of both eyes.

Sterile coupling gel was applied to the probe, which was gently apposed against the cornea with minimal pressure to the globe. Optimal positioning was confirmed when the posterior wall of the globe was clearly visible and the reflections from the 4 principal landmarks along the optical axis (cornea, anterior, and posterior lens surface and retina) were perpendicular.

Images were obtained in duplicate by 2 investigators and stored digitally. All measurements were taken from the ultrasound images and carried out using B-scan and ultrasound internal callipers. The ACD was the distance from the central cornea (maximum convexity point) to the anterior lens capsule; the CLT was the distance from the anterior lens capsule to the posterior lens capsule; the VCD was the distance from the posterior lens capsule to the retina; the AGL was the distance from the central cornea to the retina; the LGL was the distance from the lateral globe to the medial globe through the long axis of the lens; the LPD was the distance from lens poles, corresponding to the long axis of the lens. After examination, eyes were cleaned with lactated Ringer’s solution.

### Statistical analysis

The images were digitized, and all measurements were analysed using a dedicated software of the echography machine (MyLab One, ESAOTE, Genova, Italy). A blind evaluation with 2 expert investigators was performed, and intraobserver and interobserver reproducibility with a 95% confidence interval and 95% limits of agreement was estimated with Bland-Altman and Spearman methods.

Data were stratified according to gender (male and female), laterality (right and left eye), and body weight (kg). Data were stratified according to body weight, as follows: group A (n = 12) included horses weighing <48 kg (“light” foals), and group B (n = 10) included horses ≥48 kg (“heavy” foals).

All data were analysed using the Shapiro-Wilk test to determine the normality of the variables.

The influence of gender (male or female), laterality (right or left eye), and body weight on ocular measurements was analysed by the Student *t* test. Values of P<0.05 were accepted as significant for all analyses.

## Results

### Animals

All 22 foals (13 males and 9 females) met the inclusion criteria, and 44 clinically normal eyes were examined. Breeds of horses represented included 10 Thoroughbreds, 7 Saddlebreds, 3 Quarter Horses, 1 Paint, and 1 Arabian. The 13 males had a mean body weight of 46.9±9.2 kg; the 9 females had a mean body weight of 50.4±6.5 kg. Mean time of ultrasonographic examination was approximately 25–30 min.

### B-scan ultrasonographic biometry

**Figure 1:**
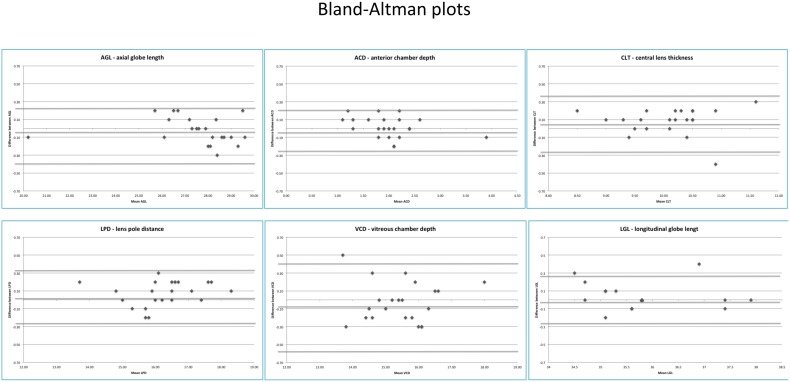
Bland-Altman plots. AGL = axial globe length; ACD = anterior chamber depth; CLT = central lens thickness; LPD = lens poles distance; VCD = vitreous chamber depth; LGL = longitudinal globe length.

**Figure 2:**
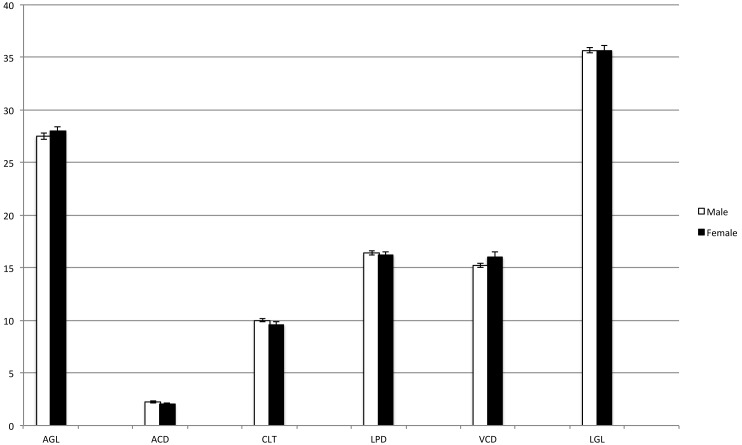
Intraocular measurements in relation to gender. No significant differences were detected. Males (white columns); females (black columns). AGL = axial globe length; ACD = anterior chamber depth; CLT = central lens thickness; LPD = lens poles distance; VCD = vitreous chamber depth; LGL = longitudinal globe length.

**Figure 3:**
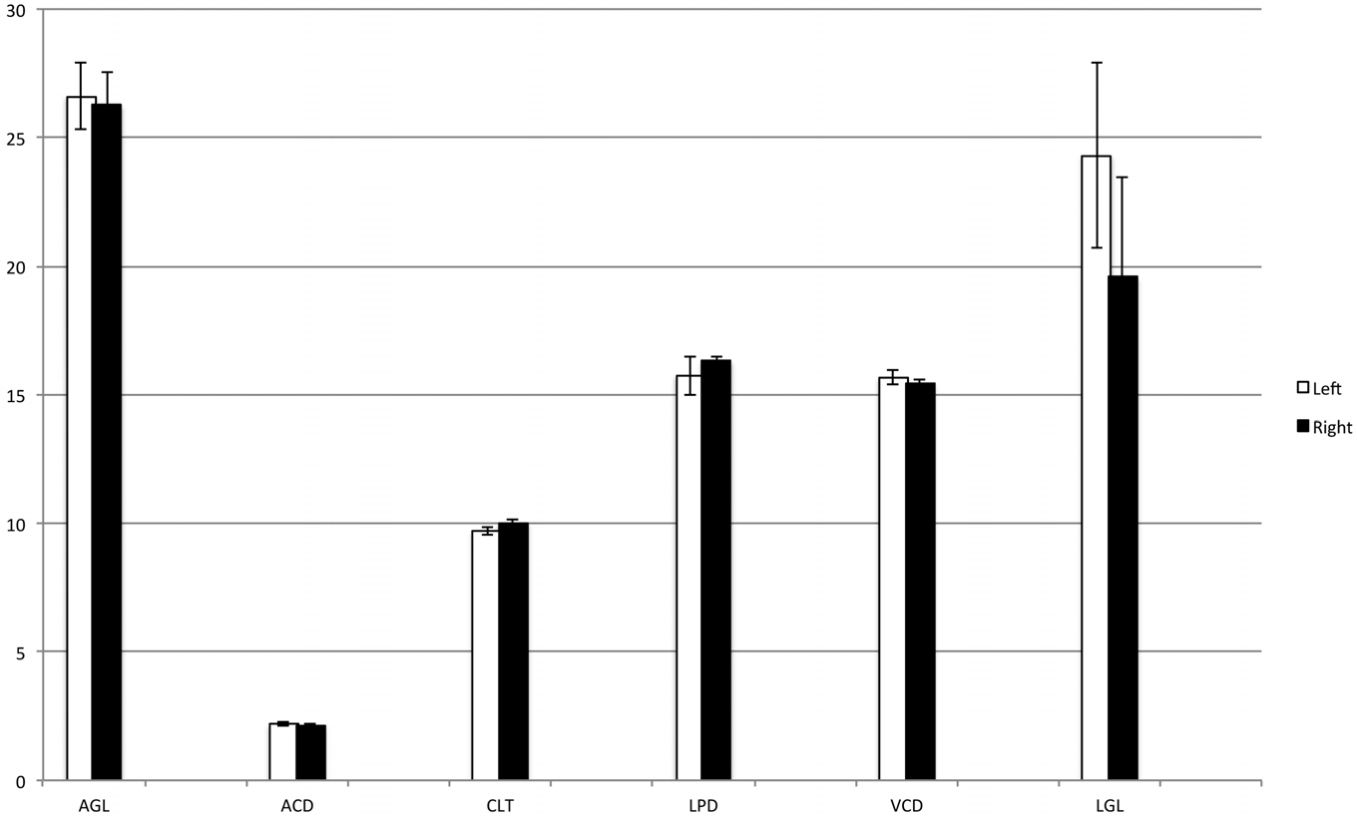
Intraocular measurements in relation to laterality. No significant differences were detected. Left eye (white columns); right eye (black columns). AGL = axial globe length; ACD = anterior chamber depth; CLT = central lens thickness; LPD = lens poles distance; VCD = vitreous chamber depth; LGL = longitudinal globe length.

**Figure 4:**
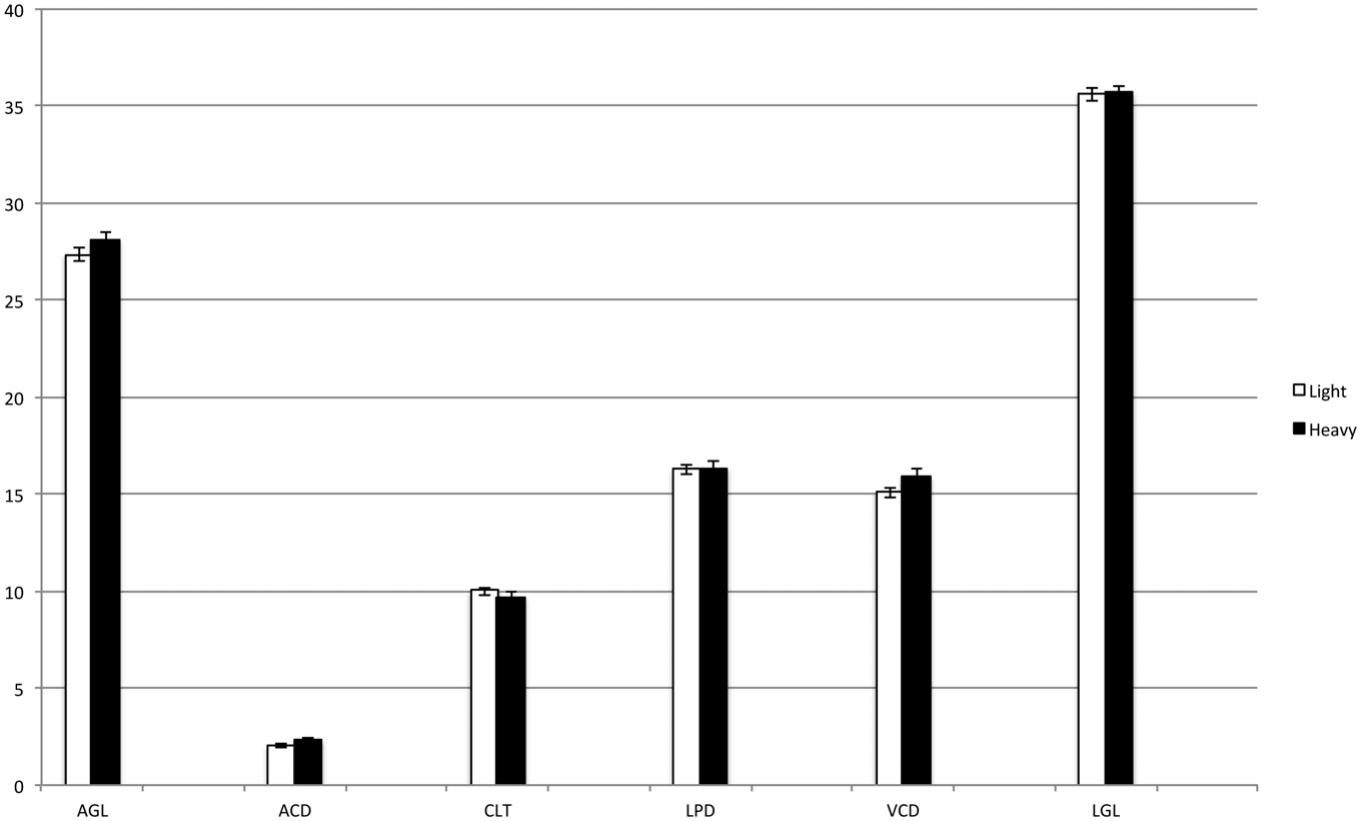
Intraocular measurements in relation to body weight. No significant differences were detected. Light <48 kg bw (wh ite columns); heavy ≥48 kg bw (black columns). AGL = axial globe length; ACD = anterior chamber depth; CLT = central lens thickness; LPD = lens poles distance; VCD = vitreous chamber depth; LGL = longitudinal globe length.

Reproducibility and agreement between operators were very good at 97% and 0.8 correlation, respectively (Fig. 1). Regarding gender, laterality, and body weight, no significant difference between males and females, between right and left eye, nor between heavier and lighter foals was detected (P<0.05) (Figs 2–4).

The mean value for ACD was 2.2±0.5 mm (SD), for CLT was 9.9±0.8 mm, for VCD was 15.5±1.1 mm, for AGL was 27.6±1.6 mm, for LGL was 35.8±1.2 mm, and for LPD was 16.4±1.0 mm.

## Discussion

In this study, the ACD, CLT, VCD, AGL, LGL, and LPD were measured in neonatal foals, and no differences were observed regarding gender, laterality, nor body weight.

A 10-MHz linear probe was used for ACD, CLT, VCD, AGL, and LPD; however, because the linear probe did not allow inclusion of the whole eye in one image, we used a microconvex probe for longitudinal globe length measurement. ACD was measured using a stand-off: the use the custom-made stand-off rather than the eyelid as a natural stand-off was preferred as it provides a lower and more homogeneous compression on the cornea and, consequently, on the anterior chamber.

As reported by Grinniger et al. [5], comparison between intraocular distances measured via A- and B-scan ultrasonography revealed a good correlation indicating that only a difference ≤1 mm existed. For this reason, A-scan biometry was not performed in our study.

To the authors’ knowledge, no previous reports describe systematic eye measurements in neonatal foals aged less than 7 days old. Renaudin et al. demonstrated a linear relationship between approximate eye volume of the foetus and day of gestation [10]; Turner et al. used ultrasonographic measures of the foetal vitreous body (length from sclera to sclera and width from retina to cornea) to estimate gestational age of the foetus in Shetland-type pony mares [11].

Ocular component measurements (including ACD, CLT, VCD, and AGL) were obtained via ultrasound in Miniature Horses aged from 1.5–264 months to establish mean values and to make comparison with values reported for full-sized adult horses. Authors concluded that Miniature Horses had smaller eyes than full-sized horses [6].

Additionally, ACD, AGL, and CLT were recorded in 14 clinically normal adult horses of various breeds via A- and B-scan ultrasound [3]; curiously, the average value of ACD (5.63±0.86) was almost comparable to that seen in Miniature Horses (mean ACD = 5.6±0.03) by Plummer et al. [6], while the other mean values were significantly higher. Those authors found that intraocular distances were shortest in the smallest horses, while greater distances were characteristic of the largest horses [3].

Grinniger et al. performed A-scan biometry on 159 horses aged from 0–30 years and B-scan biometry in 90 of those horses [5]. Thirty-three eyes were examined from the 0–2.5-year-old group, but additional information about the distribution of age within the study population was not provided; consequently, we were not able to use this study for a comparison with our data. Recently, B-mode ultrasonography was used to obtain ocular measurements in 28 adult horses [4]. Mouney et al. [4] noted that the shortest AGL was detected in the youngest horse and the longest AGL in the oldest ones, suggesting that this value depended on age rather than body size [4]. Similar considerations were made by Plummer et al. in Miniature Horses [6].

In a pilot study carried out by Townsend et al. on ocular dimensions in horses less than 1 year of age, axial dimensions were measured using A- and B-scan modalities in 10 eyes (right or left) of foals from 42–116 days old [9]. However, neonatal foals were not examined, and only means and standard deviations were reported [9].

Previous studies provided reference ranges for the ACD, CLT, VCD, and AGL in horses at or older than 1.5 months; reference ranges for LGL and LPD had never been mentioned before. With regard to laterality, our values from the right and left eyes of the foals were strongly correlated, but we cannot make comparisons with previous results because they described adult horses [3], [4], [6] or foals older than 1.5 months [9]. Both eyes were examined by Grinniger et al. but only in 6 animals of unknown age [5]; Plummer et al. compared the measurements of 2 eyes in ponies [6], but it is not possible to make correlations between data obtained by ponies and our data. Results of our study and data reported in literature were summarized in Table 1.

**Table 1:** The table compares the results of our study with intraocular measures reported in literature.

IntraocularMeasurements	Foals 1–7day-old Ourresults	Foals 42–116day-old Townsendet al., 2012 [9]	Horses 0–30YearsGrinnigeret al., 2010 [5]	Adult horsesMouney et al.,2012 [4]	Adult horsesMcMullenet al., 2006 [3]	Adult poniesPlummer et al.,2003 [6]
Anterior Chamber Depth	2,2±0,5	4,94±0,49	6,19±0,75	6,8±0,5	5,63±0,86	5,6±0,03
Central Lens Thickness	9,9±0,8	9,38±0,59	11,74±0,71	11,7±0,6	11,75±0,80	10,3±0,006
Vitreous Chamber Depth	15,5±1,1	18,96±0,86	22,98±1,86	21,8±1,3	-----------	17,8±0,18
Axial Globe Length	27,6±1,6	33,32±0,83	41,04±2,67	40,4±1,8	39,23±1,26	33,7±0,07
Longitudinal Globe Length	35,8±1,2	----------	------------	-------------	------------------	
Lens Poles Distance	16,4±1,0	----------	------------	-------------	----------------	

Values are expressed in mm and as mean value ± Standard Deviation.

Moreover, in our study, no relationship between foal gender and intraocular distances was detected, according to what has been reported in the literature for older horses [5], [6]. In adult horses, shortest intraocular distances were found in the youngest horses or those lightest in weight [4], [5]. We did not detect differences between eye measurements in light (<48 Kg) versus heavy foals (≥48 kg) (range 33–64 kg).

In conclusion, to the authors’ knowledge, this is the first study about ultrasonographic ocular measurements in neonatal foals. Our results confirmed the hypothesis that intraocular measurements were smaller in newly born foals (less than 7 days of age) compared with values reported by other authors in foals and are not associated with gender, laterality, and body weight. Limitations of this study could be the low number of foals, which should be extended to create a more reliable database. However, in spite of the number of foals examined, we believe this study provides relevant semiotic ultrasound data about intraocular measurements in neonatal foals.
